# Antigen-Specific Immunoadsorption With the Glycosorb® ABO Immunoadsorption System as a Novel Treatment Modality in Pure Red Cell Aplasia Following Major and Bidirectional ABO-Incompatible Allogeneic Hematopoietic Stem Cell Transplantation

**DOI:** 10.3389/fmed.2020.585628

**Published:** 2020-10-22

**Authors:** Ammon Handisurya, Nina Worel, Werner Rabitsch, Marija Bojic, Sahra Pajenda, Roman Reindl-Schwaighofer, Wolfgang Winnicki, Andreas Vychytil, Hanna A. Knaus, Rainer Oberbauer, Kurt Derfler, Philipp Wohlfarth

**Affiliations:** ^1^Department of Medicine III, Division of Nephrology and Dialysis, Medical University of Vienna, Vienna, Austria; ^2^1st Medical Department, Hanusch Hospital, Vienna, Austria; ^3^Department of Blood Group Serology and Transfusion Medicine, Medical University of Vienna, Vienna, Austria; ^4^Department of Medicine I, Stem Cell Transplantation Unit, Medical University of Vienna, Vienna, Austria

**Keywords:** pure red blood cell aplasia (PRCA), immunoadsorption, hematopoeietic stem cell transplantation, isohemagglutinins, Glycosorb®

## Abstract

Pure red cell aplasia (PRCA) after ABO-incompatible allogeneic hematopoietic stem cell transplantation (HSCT) is caused by persisting host-derived isohemagglutinins directed against donor red blood cell (RBC) antigens. ABO antigen-specific immunoadsorption (ABO-IA) with Glycosorb®, commonly used for desensitization therapy in ABO-incompatible living donor renal transplantation, specifically eliminates circulating isohemagglutinins and might represent a novel treatment option for post-HSCT PRCA. In this prospective observational (*n* = 3) and retrospective (*n* = 3) analysis of six adult HSCT-recipients with PRCA, ABO-IA was initiated at 159 (range: 104–186) days following HSCT. The median treatment frequency was 4.5 (range: 3.9–5.5) sessions/week. ABO-IA-treatment led to a continuous decrease in isohemagglutinin titers. Reticulocytes increased to ≥30 G/L after 17.5 (range: 4–37) immunoadsorption sessions over 28.5 (range: 6–49) days and continued to rise after that. By the end of the 3-month follow-up period after discontinuation of ABO-IA, all patients showed a sustained remission of PRCA and were independent of erythropoietin-stimulating agents and transfusions. No case of infection or graft-versus-host disease was observed. After a median follow-up of 22.03 (range: 6.08–149.00) months after ABO-IA-treatment, all patients were alive and showed a stable RBC engraftment of the donor blood group. Our data provide the first evidence for ABO-IA as an effective treatment for post-HSCT PRCA.

## Introduction

Matching of human leukocyte antigen (HLA) alleles between donors and recipients is the primary determinant of successful allogeneic hematopoietic stem cell transplantation (HSCT). Due to independent genetic inheritance, ABO mismatch between donor and recipient occurs in 30–50% of HLA-matched HSCTs but is not considered a hindrance for transplantation (1, 2). Although the influence of ABO incompatibility on the overall outcome after HSCT appears to be negligible, several associated complications such as the delayed recovery of erythropoiesis, hemolysis, or pure red cell aplasia (PRCA) have to be recognized (2, 3). The latter is related to the persistence of host B lymphocytes or plasma cells producing isohemagglutinins (IHAs) directed against donor red blood cell (RBC) antigens, leading to the disruption of erythroid hematopoiesis at the early precursor stage (2–4). Pure red cell aplasia occurs in up to 30% of all major ABO-incompatible HSCTs (4, 5). Apart from supportive measures including the administration of erythropoietin-stimulating agents (ESAs), other therapeutic approaches for PRCA include the modulation of immunosuppression, administration of immunosuppressive agents, or apheresis modalities like high-volume plasma-exchange (PE) or semi-selective immunoadsorption (5–12).

The Glycosorb® ABO immunoadsorption system is widely used for desensitization therapy in ABO-incompatible living donor renal transplantation due to its capability to remove anti-A and anti-B IHAs selectively (13, 14). In comparison to conventional antigen-unspecific immunoadsorption, ABO antigen-specific immunoadsorption (ABO-IA) using Glycosorb® might offer the benefit of effective post-HSCT PRCA treatment without the offset of affecting other blood components. In addition, immune-related complications [triggering of graft-versus-host disease (GVHD) or increased susceptibility to infections], which are associated with the use of the above mentioned alternative treatment modalities, are likely averted by Glycosorb®'s immunologically neutral mode of action (14). Here, we provide the first report available in the literature on the use of the Glycosorb® ABO immunoadsorption system to treat PRCA in six patients after HSCT.

## Materials and Methods

### Study Design and Patients

In a prospective, observational study conducted between 07/2017 and 07/2019 and an additional retrospective analysis covering the years 06/2007 until 06/2017, we analyzed data of all adult patients (*n* = 6; prospective: 3, retrospective: 3) who received immunoadsorption using Glycosorb® ABO columns for PRCA following HSCT at the Medical University of Vienna. The Institutional Review Board approved the study protocol (#EK-1953/2017), and the study was conducted following the amended Declaration of Helsinki.

### Hematopoietic Stem Cell Transplantation

Patients either received myeloablative conditioning (MAC) therapy with cyclophosphamide 120 mg/kg and 13.2 Gy hyperfractionated total body irradiation (TBI) or reduced-intensity conditioning (RIC) therapy according to the FLAMSA-RIC protocol (fludarabine 120 mg/m^2^, cytarabine 8 g/m^2^, amsacrine 400 mg/m^2^, cyclophosphamide 120 mg/kg, 4 Gy TBI). The graft source was peripheral blood stem cells (PBSC) with a target cell dose of >4 × 10^6^ CD34+ cells/kg recipient body weight administered on day 0 in all patients. Standard GVHD prophylaxis consisted of cyclosporine A (CSA) + methotrexate (MTX) following MAC or CSA + mycophenolate mofetil (MMF) following RIC. Administration, dosing, and tapering of GVHD prophylaxis followed the recommendations of the European Blood and Marrow Transplantation Society (15). Patients additionally received anti-thymocyte globulin (ATG-Fresenius) at cumulative doses of 30 mg/kg in case of a 10/10 HLA-matched unrelated donor or 60 mg/kg in case of a 9/10 HLA-matched unrelated donor. Transfusion policies regarding the use of blood groups in ABO-incompatible HSCT followed published recommendations (2). Packed RBCs and single-donor cytomegalovirus (CMV)-negative platelet concentrates were administered to maintain hemoglobin levels ≥8.0 g/dL and platelet counts ≥20 G/L, respectively. Red blood cell units were leukocyte-reduced by filtration and irradiated with 30 Gy, whereas platelet concentrates were pathogen-reduced using the intercept technology. Prophylactic RBC exchange was performed in one patient with a bidirectional ABO-mismatch undergoing RIC, as described previously (16). Chimerism analysis was performed using the Mentype® Chimera polymerase chain reaction (PCR) amplification kit (Biotype Diagnostic GmbH, Dresden, Germany).

### Pure Red Cell Aplasia

All included patients met the following diagnostic criteria for PRCA: (1) reticulocytopenia <30 G/L for more than 60 days after HSCT in association with neutrophil recovery, (2) lack of erythroblasts in the bone marrow, (3) major or bidirectional ABO mismatch between donor and recipient, and (4) exclusion of other causes for the condition (e.g., disease relapse, infection etc.).

Bone marrow biopsy, performed before ABO-IA-treatment, documented the absence of RBC precursors in all study participants. Parvovirus B19 infection and Human Herpesvirus-6 reactivation were excluded in bone marrow samples using specific PCR assays; other viral reactivations (CMV, herpes simplex virus 1/2, varicella-zoster virus, Epstein-Barr virus, adenovirus) were ruled out using PCR testing in peripheral blood samples.

All patients received transfusion support to maintain the outlined thresholds and were started on darbepoetin alfa at a weekly dose of 150 μg or epoetin theta at a weekly dose of 30,000 I.E. after diagnosis of PRCA. The tapering schedule of GVHD prophylaxis was performed per-protocol and was not altered in an attempt to treat PRCA.

### Immunoadsorption

ABO antigen-specific immunoadsorption was performed according to a standardized protocol (14). In brief, plasma was separated from whole blood by centrifugation using the COBE® SPECTRA™ (COBE Laboratories, Zaventem, Belgium; *n* = 2) or the Spectra Optia® (Terumo BCT, Lakewood, CO, USA; *n* = 4) apheresis system. Anticoagulation was performed with citrate (ACD-A, anticoagulant citrate dextrose, formula A; Baxter, Munich, Germany) and sodium heparin (Heparin Immuno, Baxter-Immuno AG, Vienna, Austria; infusion rate: 1000 IE/h). After separation, the plasma passed a Glycosorb® ABO immunoadsorption column (Glykorex Transplantation AB, Lund, Sweden), which contains synthetic terminal trisaccharides from A- or B-ABO blood group antigens bound to a sepharose matrix (14) and was then re-transfused together with the separated blood cells. Conservation and regeneration of each Glycosorb® column assigned explicitly to the same patient was performed as described previously (14).

### Measurement of Isoagglutinin Titers

Anti-IgG warm reactive and NaCl cold/room temperature reactive (IgM) titers of circulating IHAs were measured by using the Diamed ID-Card gel card technology (Bio-Rad, Munich, Germany) as described elsewhere (17).

### Data Analysis and Presentation

Data were collected from electronic patient charts. The time from HSCT to 3 months after discontinuation of ABO-IA was chosen as the individual follow-up period regarding PRCA-related outcomes. For analyzing long-term survival, the time from discontinuation of ABO-IA to the date of the last patient visit was assessed (all patients were alive at the time of manuscript submission). Engraftment was defined as the first of 3 consecutive days of an absolute neutrophil count (ANC) >500/μL. Descriptive data are presented as absolute numbers and continuous variables as median (range).

## Results

### Patient Characteristics Before Initiation of Immunoadsorption

**Figure 1:**
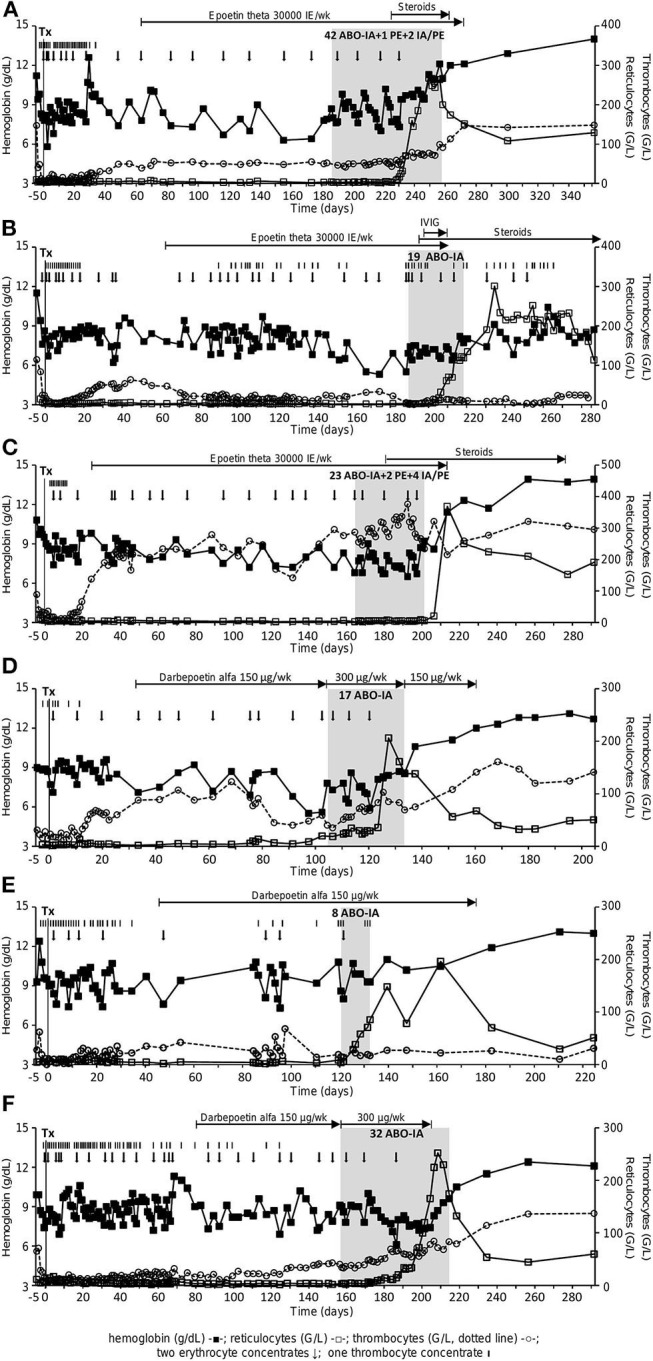
Course of hemoglobin (g/dL, full squares), reticulocytes (G/L, open squares) and thrombocytes (G/L, open circles, dotted line) in patient 1–6 **(A–F)**. Black arrows depict the administration of two packed RBCs, black bars the administration of one thrombocyte concentrate. The gray area shows the phase of treatment with ABO antigen-specific immunoadsorption. ABO-IA, ABO antigen-specific immunoadsorption; IVIG, intravenous immunoglobulins; PE, plasma exchange; Tx, transplant.

Demographic and HSCT-related data are presented in Table 1. The median reticulocyte count on day 60 after HSCT was 4.3 (2.3–5.9) G/L, and all patients were transfusion-dependent. A median number of 1.38 (0.76–1.78) packed RBCs was transfused per week between neutrophil engraftment and initiation of ABO-IA. Figure 1 shows the course of hemoglobin, reticulocytes, and thrombocytes during the individual follow-up period for each patient. Blood cell counts and other laboratory data at the time of the initiation of ABO-IA are shown in Table 2.

**Table 1:** Patient characteristics and transplant-related data.

	**All patients (*n* = 6)**
Male/Female	5/1
Age (years)	45 (27–61)
**Donor**	
10/10 MUD	5
9/10 MUD	1
**Graft source**	
PBSC	6
CD34+ (10^6^/kg)	7.09 (5.96–8.14)
**Diagnosis**	
AML	3
CML	2
MDS	1
**Conditioning regimen**	
TBI 13.2/Cy	4
FLAMSA	2
**GvHD prophylaxis**	
CSA/MTX	4
CSA/MMF	2
Anti-thymocyte globulin	6
**Engraftment and chimerism**	
Neutrophil engraftment (days after HSCT)	23 (14–28)
Platelet engraftment (days after HSCT)	24 (12–128)
Full CD3 & CD33 peripheral blood donor chimerism (days after HSCT)	28 (22–32)
**ABO mismatch** ***(recipient—donor)***	
Major	5
*0—A*	2
*0—B*	1
*0—AB*	2
Bidirectional	1
*A—B*	1

*AML, acute myeloid leukemia; CML, chronic myeloid leukemia; CSA, cyclosporine A; GvHD, graft-versus-host disease; HSCT, hematopoietic stem cell transplantation; MDS, myelodysplastic syndrome; MMF, mycophenolate mofetil; MTX, methotrexate; MUD, matched unrelated donor; PBSC, peripheral blood stem cells; TBI, total body irradiation*.

**Table 2:** Laboratory parameters prior to ABO antigen-specific immunoadsorption and immunoadsorption-related data.

	**All patients (*n* = 6)**
**Laboratory data prior to ABO-IA-treatment**	
Ferritin (μg/L)	3854.9 (2418.6–5367.5)
Transferrin (mg/dL)	164 (108–169)
Transferrin saturation (%)	86.8 (84.3–93.9)
Leukocytes (G/L)	3.02 (1.49–6.08)
Creatinine (mg/dL)	1.04 (0.80–1.23)
Bilirubin (mg/dL)	0.95 (0.35–1.64)
LDH (U/L)	191 (131–239)
CRP (mg/dL)	0.61 (0.14–3.00)
**Treatment-related data**	
Treatment modality	
Immunoadsorption only (%^*^, *n*)	94%, 21 (8–42)
Immunoadsorption plus plasma exchange (%^*^, *n*)	4%, 0 (0–4)^+^
Plasma exchange only (%^*^, *n*)	2%, 0 (0–2)^+^
Processed plasma volume	
Immunoadsorption only (mL)	11,000 (350–15,000)
Plasma exchange only (mL)	4,000 (1,000–4,700)
No. of treatments/adsorber (*n*)	1 (1–8)

+ *Performed in two of six patients*.

* *Percentage of total treatment sessions*.

Therapy with ESAs was initiated after a median of 55 (24–77) days post-HSCT but did not result in a clearance of PRCA after 14.0 (10.1–20.0) weeks of therapy. No other treatment for PRCA was initiated before ABO-IA. Tapering of immunosuppression was performed per-protocol from day 90 after HSCT onwards. No GVHD was present in any of the patients.

### Effects of ABO Antigen-Specific Immunoadsorption

Treatment with ABO-IA was started 159 (104–186) days after HSCT. A median number of 24 (8–45) treatment sessions over 33 (12–71) days were performed in each patient via peripheral venovenous access. This corresponded to an individual treatment frequency of 4.51 (3.86–5.49) sessions per week. To promote the removal of circulating IHAs, two adsorbers (anti-A with anti-A, anti-B with anti-AB, and anti-A with anti-B) were connected either in parallel or in series in one patient with a donor-to-recipient ABO mismatch of A to 0 and the two patients with an AB to A/0 mismatch in 40.9, 34.4, and 100% of treatments, respectively (Figures 1A,C,F). In two patients with prolonged treatment duration, ABO-IA was combined with PE during 4.44 and 13.79% treatment sessions, respectively, to promote the elimination of circulating IgM-IHAs. To ensure short treatment intervals, some sessions of PE only were performed in the same two patients at weekends (2.22 and 6.90% of their treatments, respectively), when ABO-IA was unavailable due to logistic reasons (Figures 1A,C, Table 2). Further treatment-related data are presented in Table 2. After initiation of treatment, one patient was diagnosed with anti-GPIIb/GPIIIa positive immune thrombocytopenia and was given intravenous immunoglobulins plus prednisone 1 mg/kg body weight (Figure 1B). Likewise, the two above mentioned patients with prolonged duration of ABO-IA-treatment were additionally treated with low-dose prednisone (0.3 mg/kg body weight) and prednisone ≥1 mg/kg body weight, respectively (Figures 1A,C).

**Figure 2:**
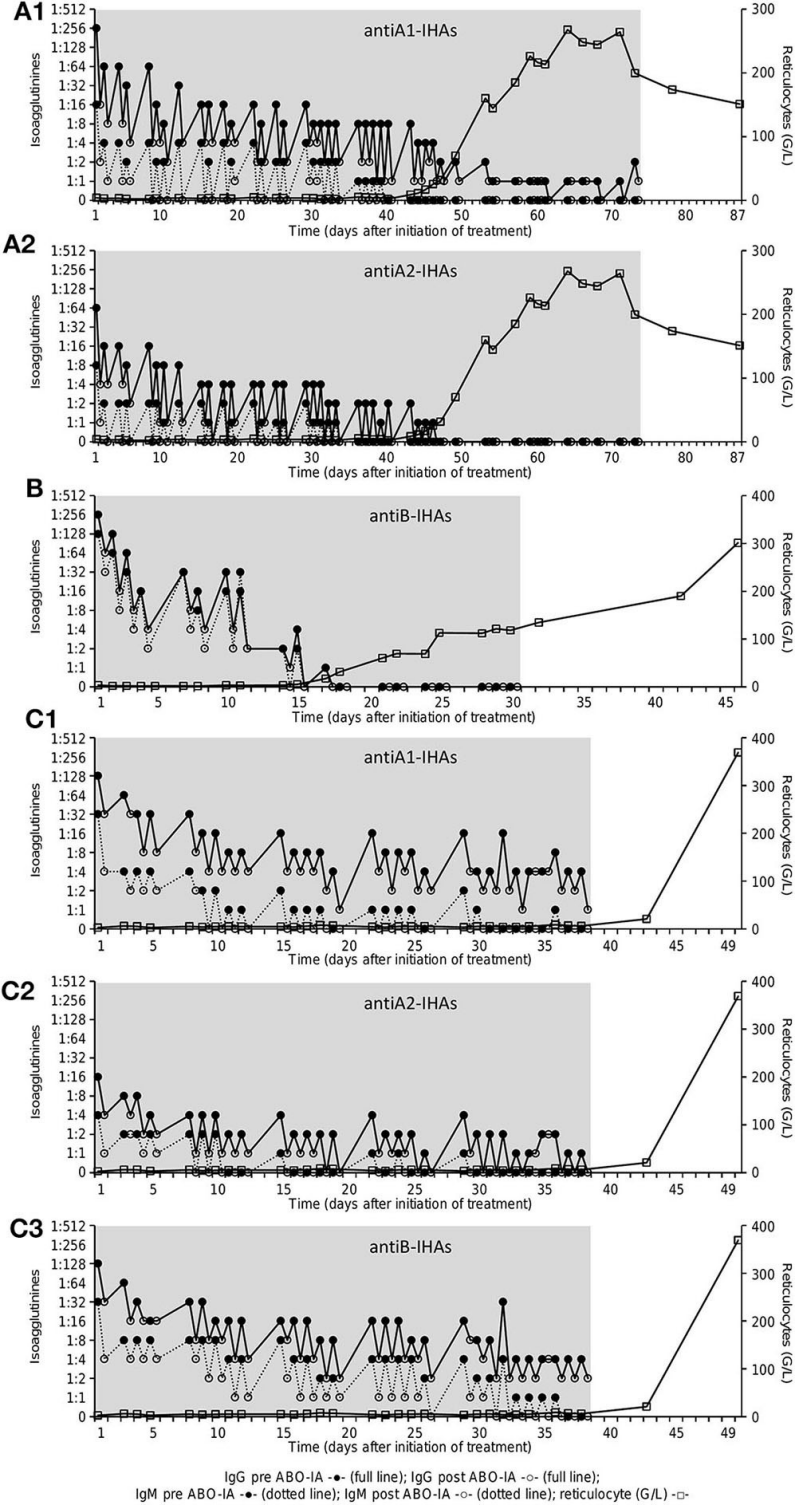
Course of IHA-titer [Coombs-IgG (full line) and NaCl/room temperature-IgM (dotted line)] before (full circles) and after (open circles) immunoadsorption as well as reticulocyte count (G/L, open squares) in patient 1–3 **(A–C)**: antiA1-IHAs **(A1)** and antiA2-IHAs **(A2)** in patient 1 **(A)**, antiB-IHAs **(B)** in patient 2 **(B)**, antiA1-IHAs **(C1)**, antiA2-IHAs **(C2)** and antiB-IHAs **(C3)** in patient 3 **(C)**. The gray area shows the treatment period with ABO antigen-specific immunoadsorption. IHA, isohemagglutinins.

Data on IgG- and IgM-IHA titers were available for the three patients enrolled in the prospective protocol and showed a continuous decrease during ABO-IA-treatment (Figure 2). Reticulocyte counts increased to ≥30 G/L after a median of 17.5 (4–37) treatments and after 28.5 (6–49) days from the initiation of ABO-IA. ABO antigen-specific immunoadsorption was discontinued in 5/6 (83%) patients when reticulocyte counts increased to levels ≥80 G/L, suggesting the resolution of PRCA (Figure 1). Only one patient showed a reticulocyte count of 6.2 G/L, when ABO-IA was stopped due to logistic reasons (Figure 1C). However, in this patient, reticulocyte counts started to rise 2 days after the last ABO-IA and reached >360 G/L within the next 2 weeks, strongly suggesting a positive response to the treatment.

In addition to PRCA, five (83%) patients also presented with distinct thrombocytopenia (≤50 G/L, Figure 1) when ABO-IA-therapy was initiated. In thrombocytopenic patients (*n* = 5), ABO-IA-treatment resulted in an increase of thrombocyte counts from 40 (1–50) to 69 (7–91) G/L at the time of the final ABO-IA.

No side effects attributed to ABO-IA were observed during or after any of the procedures. One patient developed an anaphylactic reaction during PE, presumably caused by frozen plasma units (Octaplas) as substitution fluid. All ABO-IA-sessions were conducted according to schedule, and none had to be aborted due to patient-related complications apart from rarely occurring treatment interruptions due to cannula dislocations.

### Follow-Up

All patients achieved remission of PRCA and sustained transfusion independence during the individual follow-up period. Only one patient, who developed an immune thrombocytopenia, received RBCs due to episodes of gastrointestinal bleeding on three time-points within the first month after the last ABO-IA (reticulocyte counts remained high, Figure 1B). The median hemoglobin and reticulocyte levels at 30 and 90 days after discontinuation of ABO-IA were 11.6 (7.9–12.9) g/dL and 152.0 (53.5–227.1) G/L, and 13.0 (8.9–14.0) g/dL and 94.2 (50.0–191.8) G/L, respectively (Figure 1). During the individual follow-up, thrombocyte counts continued to rise to a median of 137 (28–148) G/L in the five patients, who were thrombocytopenic at the time of initiation of ABO-IA. Importantly, no cases of GVHD or infections requiring systemic antimicrobial therapy were documented during ABO-IA-treatment and the first 3 months after the last ABO-IA. Conversion to the donor blood group was observed in all patients. All patients were alive after a median follow-up of 22.03 (6.08–149.00) months after discontinuation of ABO-IA.

## Discussion

Our data demonstrate the successful resolution of PRCA in six major/bidirectional ABO-incompatible allogeneic HSCT recipients by ABO-IA using the Glycosorb® ABO immunoadsorption system. Of note, the time between initiation of ABO-IA and the recovery from PRCA was short, and no cases of GVHD or infection occurred during the treatment phase or the follow-up period of 3 months after the last treatment.

To date, conflicting results exist concerning the natural course of PRCA after HSCT. While PRCA may resolve spontaneously in some patients receiving supportive measures only (administration of ESAs), others need specific treatment (18). The probability of a spontaneous remission is low, if IHAs directed against donor RBCs persist for more than 60 days (2). One strategy to overcome PRCA is the tapering of immunosuppression to elicit a graft-mediated immune effect against residual IHA producing B- and plasma cells of the host (4, 18, 19). This approach has been reported effective in several cases but poses the patient at a significant risk of developing GVHD, which can be a fatal complication (20). Other strategies use immunosuppressive agents, including high-dose steroids, rituximab, bortezomib or daratumumab, in an attempt to suppress the production of anti-donor IHAs or employ antigen-unspecific apheresis modalities to remove circulating host IHAs (5–12).

Of the strategies mentioned above, the chimeric monoclonal anti-CD20 antibody rituximab is currently among the most widely applied with several reports indicating its efficacy (6, 11). However, not all patients respond with a complete resolution of PRCA to its use (9–11). In addition, rituximab may cause infusion reactions and substantially increases the susceptibility to infections due to a long-lasting B-cell depletion (21). While the detailed pathophysiological background of PRCA after major ABO-incompatible HSCT is not fully elucidated, it is well-acknowledged that host IHAs directed against donor RBCs are responsible for the development of PRCA (4). Interestingly, Griffith et al. (22) provided evidence that host plasma cells, in which the CD20-expression is down-regulated, rather than host B cells are the primary source of anti-donor IHAs, which explains the resistance of at least some cases of PRCA to rituximab treatment.

In contrast to rituximab, apheresis modalities, including PE and immunoadsorption, do not inhibit or eliminate antibody-producing cells but rapidly remove circulating IHAs. The feasibility of both apheresis modalities in the treatment of PRCA following major ABO-incompatible HSCT has previously been demonstrated (5–8). However, the few published studies on the effects of immunoadsorption on PRCA all used antigen-unspecific immunoglobulin-adsorbers. While these adsorbers and, notably, also PE non-specifically remove IgG and IgM from the circulation, the Glycosorb® ABO immunoadsorption columns specifically bind and remove anti A- and/or anti B-IHAs only. As a result, the pool of remainder immunoglobulins is preserved and the humoral immune response remains unimpaired (17). This is an important aspect in the HSCT setting, as infections are a significant contributor to morbidity and mortality in the early post-transplant phase (23, 24). Indeed, no episodes of infection or GVHD were documented in our patients during ABO-IA and the 3-month follow-up period thereafter. Adding to the favorable adsorption profile, the Glycosorb® ABO antigen-specific adsorbers were previously shown to be superior in terms of blood group-specific IgM removal as compared to semi-selective adsorbers based on staphylococcal protein A, synthetic peptide GAM146 or immobilized polyclonal sheep antibodies directed at human immunoglobulin in ABO-incompatible kidney transplant (17, 25).

In general, immunoadsorption may be more efficient than PE in removing circulating antibodies due to the higher plasma volume processed during a single session. Evidence for this predication in PRCA following major ABO-incompatible HSCT was previously provided by Rabitsch et al. (7), who successfully treated two patients non-responding to PE with immunoadsorption. Further, immunoadsorption lacks potential side effects conferred by PE, including electrolyte disturbances, anaphylactic reactions caused by plasma as replacement fluid, and, of note, bleeding complications by the depletion of fibrinogen and other coagulation factors (26). The latter may especially be relevant in HSCT recipients with PRCA, as these patients often additionally feature profound thrombocytopenia (27). Indeed, at the time of ABO-IA initiation, five out of six patients in our cohort presented with distinct thrombocytopenia, which significantly improved following ABO-IA. ABO antigens are not exclusively expressed on the surface of RBC but also on platelets, which may also absorb soluble A and B antigens from plasma (27). Thus, the successful removal of IHAs may have accounted for the rising thrombocyte counts in some of our patients. In contrast, one patient with immune thrombocytopenia did not respond to the treatment with ABO-IA, as the detected GPIIb/GPIIIa antibodies are not bound by the Glycosorb® columns.

In our study, ABO-IA was discontinued after clinical response by an increase in reticulocyte counts to levels ≥80 G/L could be documented. This decision was based on findings of our previous work, demonstrating sustained remissions without documented cases of PRCA relapse following IHA clearance by conventional immunoadsorption (7, 8). The mechanism of these persistent remissions is unclear. However, it may be hypothesized that the reduction of the IHA titers allows for breakthrough hematopoiesis to occur, which leads to scavenging of residual antibodies and immunological tolerance by excess donor RBCs and ABO antigen. Notably, the median time between the initiation of ABO-IA and the increase of reticulocyte counts was short (median of 28.5 days to reach ≥30 G/L), corroborating the highly efficient elimination of IHAs as demonstrated in three patients with available serial IHA measurements (Figure 2). Previous studies on the successful treatment of PRCA after major ABO-incompatible HSCT reported on a time to response of 16–204 days using PE or antigen-unspecific immunoadsorption (5), 21–49 days for rituximab (11), and up to 16 months using a combination of different therapies (6), respectively. Differences in the treatment schedule as well as other confounders (3, 4) may have accounted for the observed variation in response times in our as well as other studies using immunoadsorption or PE. In this context, longer intervals between each treatment session may cause a rebound of circulating IHAs (28) and should therefore be kept short.

A major shortcoming of the Glycosorb® ABO immunoadsorption columns are the high costs when each adsorber is used only once, as recommended by the manufacturer (€ 3932 for a single use including all disposables and personnel time at our center). A recent study in 71 ABO-incompatible renal transplant recipients, however, provided evidence for an efficient and safe re-use of the columns (13). In our study, up to eight ABO-IA-treatments were safely conducted with one adsorber in the same patient. Despite the significant reduction of treatment-related expenses by this approach, the costs considerably exceed those of semi-selective immunoadsorption or PE (29) and also of pharmacological treatment options like rituximab (30). As a comparison, the costs of PE with 4 L of human albumin, including disposables and personnel costs, amount to € 1194 at our center (29) but can increase significantly, if fresh frozen plasma is used as substitution fluid to prevent bleeding in thrombocytopenic patients (26).

Our study has several limitations. First, the number of included patients is small. However, this also applies to most other studies reporting on post-HSCT PRCA directed treatments, which likely reflects the rarity of this complication. Second, this is an observational study and, thus, patients were not treated on a uniform treatment protocol. Treatment frequencies and adsorber configurations accordingly varied between patients. Third, patients were treated in a center with a dedicated facility and high-level experiences in available methods of PE and immunoadsorption. The Glycosorb® adsorbers are mainly used for desensitization therapy in ABO-incompatible living donor renal transplantation and ABO-IA may, thus, not be available in centers exclusively performing HSCT. However, ABO-IA using the Glycosorb® ABO immunoadsorption system can be performed with devices for plasma separation as used in commonly available PE and does not require devices for adsorber loading and desorption like for semi-selective immunoadsorption.

In conclusion, our data provide the first evidence for ABO-IA using Glycosorb® ABO immunoadsorption columns as an efficient and safe therapeutic option for the treatment of PRCA after ABO-incompatible allogeneic HSCT. Of note, ABO-IA—in contrast to other treatment modalities—does not confer an increased susceptibility to infectious complications or the development of GVHD and may be more efficient in the removal of circulating IHAs than other apheresis modalities.

## Data Availability Statement

The raw data supporting the conclusions of this article will be made available by the authors, without undue reservation.

## Ethics Statement

The studies involving human participants were reviewed and approved by Ethics Committee of the Medical University of Vienna. The patients/participants provided their written informed consent to participate in this study.

## Author Contributions

AH and PW: wrote the manuscript, data calculation, and data assessment. NW, AV, and HK: data assessment and substantial contribution to the discussion. WR: project design, data interpretation, and substantial contribution to the discussion. MB, SP, and RR-S: project design, data assessment, and data interpretation. WW: substantial contribution to the discussion. RO: project design and substantial contribution to the discussion. KD: project design, data assessment, substantial contribution to the discussion. All authors contributed to the article and approved the submitted version.

## Conflict of Interest

The authors declare that the research was conducted in the absence of any commercial or financial relationships that could be construed as a potential conflict of interest.

## Abbreviations

ABO-IA: ABO antigen-specific immunoadsorption
CMV: cytomegalovirus
CSA: cyclosporine A
EBMT: European Blood and Marrow Transplantation Society
ESA: erythropoietin-stimulating agents
HLA: human leukocyte antigen
GVHD: graft-versus-host disease
HSCT: hematopoietic stem cell transplantation
IHA: isohemagglutinine
MAC: myeloablative conditioning
MMF: mycophenolate mofetil
MTX: methotrexate
MUD: matched unrelated donor
PCR: polymerase chain reaction
PBSC: peripheral blood stem cells
PE: plasma exchange
PRCA: pure red cell aplasia
RBC: red blood cell
RIC: reduced-intensity conditioning
TBI: total body irradiation.

## References

[1] Damodar S Shanley R MacMillan M Ustun C Weisdorf D. Donor-to-recipient ABO mismatch does not impact outcomes of allogeneic hematopoietic cell transplantation regardless of graft source. Biol Blood Marrow Transplant. (2017) 23:795–804. 10.1016/j.bbmt.2017.02.009 28232088 PMC5744261

[2] Worel N. ABO-mismatched allogeneic hematopoietic stem cell transplantation. Transfus Med Hemother. (2016) 43:3–12. 10.1159/000441507 27022317 PMC4797460

[3] Rowley SD Donato ML Bhattacharyya P Red blood cell-incompatible allogeneic hematopoietic progenitor cell transplantation. Bone Marrow Transplant. (2011) 46:1167–85. 10.1038/bmt.2011.135 21897398

[4] Bolan CD Leitman SF Griffith LM Wesley RA Procter JL Stroncek DF. Delayed donor red cell chimerism and pure red cell aplasia following major ABO-incompatible nonmyeloablative hematopoietic stem cell transplantation. Blood. (2001) 98:1687–94. 10.1182/blood.V98.6.1687 11535498

[5] Worel N Greinix HT Schneider B Kurz M Rabitsch W Knöbl P. Regeneration of erythropoiesis after related- and unrelated-donor BMT or peripheral blood HPC transplantation: a major ABO mismatch means problems. Transfusion. (2000) 40:543–50. 10.1046/j.1537-2995.2000.40050543.x 10827256

[6] Helbig G Stella-Holowiecka B Wojnar J Krawczyk M Krzemien S Wojciechowska-Sadus M. Pure red-cell aplasia following major and bi-directional ABO-incompatible allogeneic stem-cell transplantation: recovery of donor-derived erythropoiesis after long-term treatment using different therapeutic strategies. Ann Hematol. (2007) 86:677–83. 10.1007/s00277-007-0304-8 17486341

[7] Rabitsch W Knöbl P Prinz E Keil F Greinix H Kalhs P. Prolonged red cell aplasia after major ABO-incompatible allogeneic hematopoietic stem cell transplantation: removal of persisting isohemagglutinins with Ig-Therasorb immunoadsorption. Bone Marrow Transplant. (2003) 32:1015–19. 10.1038/sj.bmt.1704264 14595389

[8] Rabitsch W Knöbl P Greinix H Prinz E Kalhs P Hörl WH. Removal of persisting isohaemagglutinins with Ig-Therasorb immunoadsorption after major ABO-incompatible non-myeloablative allogeneic haematopoietic stem cell transplantation. Nephrol Dial Transplant. (2003) 18:2405–8. 10.1093/ndt/gfg364 14551374

[9] Shahan JL Hildebrandt GC. Successful treatment of refractory pure red cell aplasia with bortezomib after allogeneic haematopoietic cell transplantation in a patient with alpha-beta subcutaneous panniculitis-like T celllymphoma. Transfus Med. (2015) 25:342–4. 10.1111/tme.12216 26147536

[10] Chapuy CI Kaufman RM Alyea EP Connors JM. Daratumumab for delayed red-cell engraftment after allogeneic transplantation. N Engl J Med. (2018) 379:1846–50. 10.1056/NEJMoa1807438 30403942

[11] Zhidong W Hongmin Y Hengxiang W. Successful treatment of pure red cell aplasia with a single low dose of rituximab in two patients after major ABO incompatible peripheral blood allogeneic stem cell transplantation. Transfus Med. (2012) 22:302–4. 10.1111/j.1365-3148.2012.01156.x 22540210

[12] Deotare UR Vishwabandya A Mathews V George B Srivastava A Chandy M. Response to high-dose dexamethasone for acquired pure red cell aplasia following ABO-mismatched allogeneic stem cell transplantation. Bone Marrow Transplant. (2006) 37:1149–50. 10.1038/sj.bmt.1705378 16699532

[13] Schiesser M Steinemann DC Hadaya K Huynh-Do U Eisenberger U Binet I. The reuse of immunoadsorption columns in ABO-incompatible kidney transplantation is efficient: the swiss experience. Transplantation. (2015) 99:1030–5. 10.1097/TP.0000000000000457 25340594

[14] Kumlien G Ullström L Losvall A Persson LG Tydén G. Clinical experience with a new apheresis filter that specifically depletes ABO blood group antibodies. Transfusion. (2006) 46:1568–75. 10.1111/j.1537-2995.2006.00927.x 16965585

[15] Penack O Marchetti M Ruutu T Aljurf M Bacigalupo A Bonifazi F. Prophylaxis and management of graft versus host disease after stem-cell transplantation for haematological malignancies: updated consensus recommendations of the European Society for Blood and Marrow Transplantation. Lancet Haematol. (2020) 7:e157–67. 10.1016/S2352-3026(19)30256-X 32004485

[16] Worel N Greinix HT Supper V Leitner G Mitterbauer M Rabitsch W. Prophylactic red blood cell exchange for prevention of severe immune hemolysis in minor ABO-mismatched allogeneic peripheral blood progenitor cell transplantation after reduced-intensity conditioning. Transfusion. (2007) 47:1494–502. 10.1111/j.1537-2995.2007.01289.x 17655594

[17] Wahrmann M Schiemann M Marinova L Körmöczi GF Derfler K Fehr T. Anti-A/B antibody depletion by semiselective versus ABO blood group-specific immunoadsorption. Nephrol Dial Transplant. (2012) 27:2122–9. 10.1093/ndt/gfr610 22086972

[18] Aung FM Lichtiger B Bassett R Liu P Alousi A Bashier Q. Incidence and natural history of pure red cell aplasia in major ABO-mismatched haematopoietic cell transplantation. Br J Haematol. (2013) 160:798–805. 10.1111/bjh.12210 23330820 PMC4078723

[19] Yamaguchi M Sakai K Murata R Ueda M. Treatment of pure red cell aplasia after major ABO-incompatible peripheral blood stem cell transplantation by induction of chronic graft-versus-host disease. Bone Marrow Transplant. (2002) 30:539–41. 10.1038/sj.bmt.1703699 12379896

[20] El-Jawahri A Li S Antin JH Spitzer TR Armand PA Koreth J. Improved treatment-related mortality and overall survival of patients with grade IV acute GVHD in the modern years. Biol Blood Marrow Transplant. (2016) 22:910–8. 10.1016/j.bbmt.2015.12.024 26748160

[21] Nixon A Ogden L Woywodt A Dhaygude A. Infectious complications of rituximab therapy in renal disease. Clin Kidney J. (2017) 10:455–60. 10.1093/ckj/sfx038 28852481 PMC5570071

[22] Griffith LM McCoy JP Jr. Bolan CD Stroncek DF Pickett AC Linton GF. Persistence of recipient plasma cells and anti-donor isohaemagglutinins in patients with delayed donor erythropoiesis after major ABO incompatible non-myeloablative haematopoietic cell transplantation. Br J Haematol. (2005) 128:668–75. 10.1111/j.1365-2141.2005.05364.x 15725089

[23] Hirokawa M Fukuda T Ohashi K Hidaka M Ichinohe T Iwato K. Efficacy and long-term outcome of treatment for pure red cell aplasia after allogeneic stem cell transplantation from major ABO-incompatible donors. Biol Blood Marrow Transplant. (2013) 19:1026–32. 10.1016/j.bbmt.2013.04.004 23583828

[24] Mikulska M Del Bono V Bruzzi P Raiola AM Gualandi F Van Lint MT. Mortality after bloodstream infections in allogeneic haematopoietic stem cell transplant (HSCT) recipients. Infection. (2012) 40:271–8. 10.1007/s15010-011-0229-y 22187340

[25] Thölking G Koch R Pavenstädt H Schuette-Nuetgen K Busch V Wolters H. Antigen-specific versus non-antigen-specific immunoadsorption in ABO-incompatible renal transplantation. PLoS ONE. (2015) 10:e0131465. 10.1371/journal.pone.0131465 26121389 PMC4488147

[26] Zöllner S Pablik E Druml W Derfler K Rees A Biesenbach P. Fibrinogen reduction and bleeding complications in plasma exchange, immunoadsorption and a combination of the two. Blood Purif . (2014) 38:160–6. 10.1159/000367682 25501972

[27] Aung FM Lichtiger B Rondon G Yin CC Alousi A Ahmed S. Pure red cell aplasia in major ABO-mismatched allogeneic hematopoietic stem cell transplantation is associated with severe pancytopenia. Biol Blood Marrow Transplant. (2016) 22:961–5. 10.1016/j.bbmt.2016.02.008 26921820 PMC7176024

[28] Wilpert J Geyer M Teschner S Schaefer T Pisarski P Schulz-Huotari C. ABO-incompatible kidney transplantation-proposal of an intensified apheresis strategy for patients with high initial isoagglutinine titers. J Clin Apher. (2007) 22:314–22. 10.1002/jca.20153 18095303

[29] Biesenbach P Kain R Derfler K Perkmann T Soleiman A Benharkou A. Long-term outcome of anti-glomerular basement membrane antibody disease treated with immunoadsorption. PLoS ONE. (2014) 31:e103568. 10.1371/journal.pone.0103568 PMC4117516 25079220

[30] Kim M Martin ST Townsend KR Gabardi S. Antibody-mediated rejection in kidney transplantation: a review of pathophysiology, diagnosis, and treatment options. Pharmacotherapy. (2014) 34:733–44. 10.1002/phar.1426 24753207

